# Supplementary material for: Relational models theory: Validation and replication for four fundamental relationships
Source: PLoS One. 2023 Jun 16;18(6):e0287391. doi: 10.1371/journal.pone.0287391 (PMC10275437; doi:10.1371/journal.pone.0287391)
Supplement: S1 Appendix — (DOCX) [file pone.0287391.s001.docx]

**Appendix**

*Items retained in final relational models scale, validated in Studies 2 and 3*

| Item | Wording |
| --- | --- |
| CS1 | If either of you needs something, the other gives it without expecting anything in return |
| CS5 | You make decisions together by consensus |
| CS6 | The two of you tend to develop very similar attitudes and values |
| CS7 | You feel that you have something unique in common that makes you two essentially the same |
| CS8 | The two of you are a unit: you belong together |
| AR2 | One of you is entitled to more than the other |
| AR3 | One of you directs the work you do together—the other pretty much does what they are told to do |
| AR5 | One of you makes the decisions and the other generally goes along |
| AR6 | One of you is the leader, the other loyally follows their will |
| AR8 | One of you is above the other in a kind of hierarchy |
| EM2 | You typically divide things up into shares that are the same size |
| EM3 | If you have work to do, you usually split it evenly |
| EM6 | If one person does what the other wants, next time the second person should do what the first person wants |
| EM8 | Both of you should have even chances |
| EM9 | If you can’t divide something up, you take turns |
| MP1 | What you get from this person is directly proportional to how much you give them |
| MP2 | You divide things up according to how much each of you has paid or contributed |
| MP3 | If one of you worked for the other, they would be paid in proportion to how long they worked or how much they did |
| MP4 | You have a right (you are entitled) to a fair rate of return for what you put into this interaction |
| MP7 | You expect to get the same rate of return on your effort and investment that other people get |
